# Fatal maternal complication due to neurofibromatosis type 1-associated giant pigmented plexiform neurofibromas in pregnancy: a case report and literature review

**DOI:** 10.4314/ahs.v22i1.20

**Published:** 2022-03

**Authors:** Leo Odongo, Matthias Goebeler, Hermann Kneitz, John C Lule, Godwin Turyasingura

**Affiliations:** 1 Department of Dermatology, Kabale University School of Medicine, Kabale, Uganda; 2 Department of Dermatology, Venereology and Allergology, University Hospital Würzburg, Würzburg, Germany; 3 Department of Obstetrics and Gynaecology, Kabale University School of Medicine, Kabale, Uganda

## Introduction

Neurofibromatosis type 1 (NF1) is an autosomal-dominant hereditary multi-system disorder that is associated with the development of nervous system tumours. Its incidence is estimated at 1 in 2500 newborns^1^,^2^. Clinical findings in NF1 include café-au-lait spots, cutaneous neurofibromas, axillary and inguinal freckling, iris hamartomas (Lisch nodules) and skeletal deformities^3^.

According to the diagnostic criteria for NF1 developed by the National Institutes of Health^4^, at least two of the following seven criteria are required for the diagnosis of NF1: Six or more café-au-lait macules >5 mm in greatest diameter in prepubertal individuals and >15 mm in greatest diameter in postpubertal individuals; two or more neurofibromas of any type or one plexiform neurofibroma; freckling in the axillary or inguinal regions ; optic glioma; two or more Lisch nodules (iris hamartomas); a distinctive osseous lesion such as sphenoid dysplasia or tibial pseudarthrosis; a first-degree relative diagnosed with NF1.

Plexiform neurofibromas develop in up to 50% of individuals suffering from NF1. Melanotic neurofibromas are rare tumours representing less than 1% of all neurofibromas.^5^ Remarkable courses of disease have been observed during pregnancy: Neurofibromas may increase in size or number during pregnancy^6^, potentially undergo malignant transformation,^7^,^8^ recur in a subsequent pregnancy,^9^ or may be associated with fatal maternal pregnancy outcomes^3^,^9^. We here present a case of fatal maternal outcome of melanotic NF1 in pregnancy without evidence of malignant transformation.

## Case description

**Figure 1:**
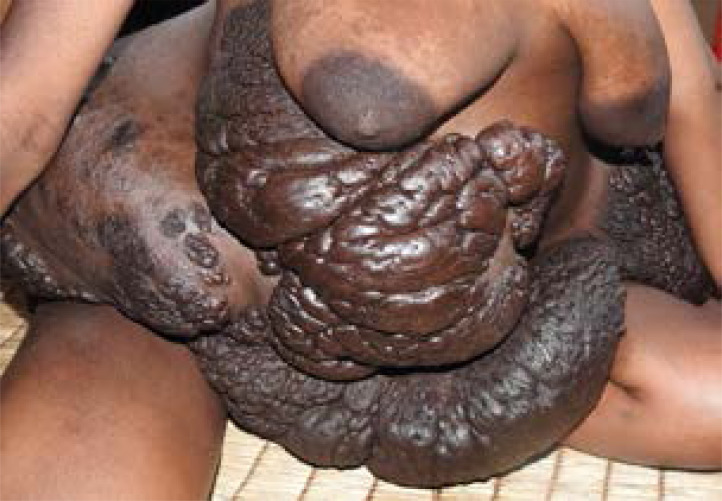
Giant plexiform neurofibromas 11 weeks after delivery of a healthy child

A 27-year-old gravida 2 para 1 Ugandan woman was admitted to the labour ward at 40 weeks of gestation in labour pains. Her first pregnancy reportedly proceeded without increase in size of the cutaneous masses -which were present before the first pregnancy- and ended in successful caesarean delivery of a healthy term baby three years before. She reported progressive increase in cutaneous trunk masses starting from the third trimester of her second pregnancy. Whereas she reportedly lost both parents during her early childhood, she reported no history of neurofibromatosis in her family. On physical examination she was normotensive and presented with café-au-lait macules, axillary freckling, kyphosis and large soft diffuse hyperpigmented plexiform neurofibromas involving the trunk. A clinical diagnosis of NF1 was made. Due to contracted pelvis her child was delivered by caesarean section. Accessing the abdominal cavity required incision of a large suprapubic plexiform neurofibroma, which offered less resistance to incision compared to normal adjacent tissues. The cut surfaces of the neurofibroma exuded with a mixture of blood and clear fluid. A healthy baby was delivered with neither intraoperative nor postoperative maternal complications. One week later, mother and child were discharged. At discharge both the newborn and the then 3-year-old sibling had no dermatological features of NF1. While at home the patient reported rapidly progressing size of cutaneous masses causing difficulty in breathing and inability to walk or lie down (Figure 1).

**Figure 2:**
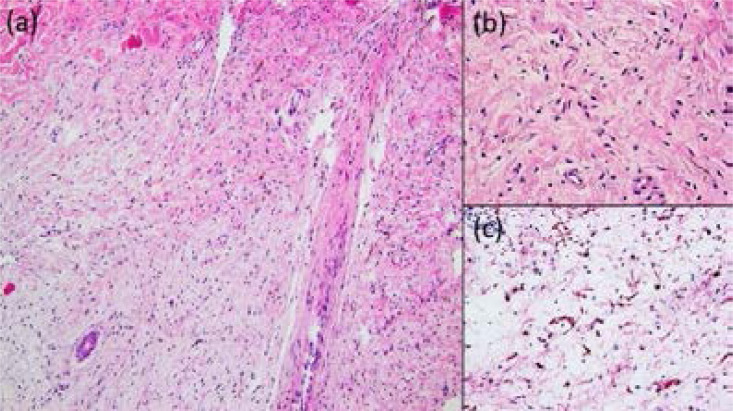
(a) Histopathologic examination showing neurofibromatous tissue with spindle-shaped tumor cells. H&E staining (×10). (b) High-power-view: Monomorphous spindle cells with elongated and wavy nucleoli, mast cells, and pigmented dendritic cells. (c) Immunohistochemical stain for S-100 protein (×40).

During a home visit 11 weeks after delivery, skin biopsies were obtained from the cutaneous masses. Histopathologic examination revealed neurofibromatous tissue with diffuse infiltrative growth pattern and monomorphic spindle cells with scarce cytoplasm showing positivity for protein S100, confirming the diagnosis of pigmented diffuse type cutaneous neurofibroma without evidence for malignant transformation (Figure 2a-c). Thirteen weeks after delivery, the patient died due to massive progress of giant plexiform neurofibromas.

## Discussion and review of literature

We here report on a 27-year-old female who died shortly after her second pregnancy due to the massive progress of giant plexiform neurofibromas. Our case shows similarities to several previous reports of neurofibromatosis-associated maternal complications of pregnancy. Isikoglu et al^10^ reported enlargement of a plexiform neurofibroma in a woman with postpartum regression in all her six pregnancies while Williams S^11^ observed enlargement of a plexiform neurofibroma in a second pregnancy without histologic evidence of malignant transformation. Whereas Posma et al^9^ and Nelson et al^3^ reported fatal maternal complications due to malignant transformation of neurofibromas, in our case there was no histopathologic evidence of transformation to malignant peripheral nerve sheath tumours. Our case disagrees with the case report by Niesert et el^12^ and the recent study by Well et al^13^ who stated that pregnancy is not significantly affecting the growth dynamics of neurofibroma. As evidenced by published cases^3^,^6^,^7^,^8^,^9^,^10^,^11^ and the report of appearance of neurofibromas for the first time during pregnancy by Xiong et al^14^, we believe that pregnancy hormones may influence growth dynamics of neurofibromas.

In accordance with Leppävirta et al^15^, we encourage intensified interdisciplinary monitoring of every pregnant woman with NF1 for possible development of complications. While MR imaging was not available for our patient, we believe that she died because of locally destructive growth of neurofibromas into ambient tissues. It might be speculated whether our patient would have had a better prognosis if we had resected the large plexiform neurofibromas as there are reports of successful surgical treatment of neurofibromas including the melanotic type^16^,^17^. In future such patients as ours could benefit from medical therapy as there are emerging encouraging reports from clinical trials with the MEK inhibitor selumetinib in the treatment of inoperable NF1-related plexiform neurofibromas^18^,^19^.

## Conclusion

The simultaneous occurrence of NF1 and pregnancy should warrant close interdisciplinary monitoring of the mother for possible complications. Even when previous pregnancies were not fatally complicated by NF1, subsequent pregnancies can still develop fatal NF1-related complications. In the absence of evidence of malignant transformation, maternal rapid and fatal progression of melanotic NF1 in pregnancy is still a possibility. Pregnancy hormones may influence growth dynamics of neurofibromas. The presence of large plexiform neurofibromas on the abdominal wall should not be an anatomical/surgical limitation of incision for accessing the abdominal cavity.
